# Fibrome En Pastille: A Case Report

**DOI:** 10.7759/cureus.116287

**Published:** 2026-09-15

**Authors:** Ramachandra Reddy Gowda Venkatesha, Karthik Rajaram Mohan, Saramma Mathew Fenn, Kumar Appusamy, Reethika Rathan

**Affiliations:** 1 Oral Medicine and Radiology, Vinayaka Mission's Sankarachariyar Dental College, Vinayaka Mission's Research Foundation (DU), Salem, IND; 2 Oral Medicine and Radiology, Vinayaka Mission’s Sankarachariyar Dental College, Vinayaka Mission’s Research Foundation (DU), Salem, IND

**Keywords:** asymptomatic presentation, dorsum of the tongue, exophytic nodule, tongue mass, traumatic fibroma

## Abstract

Traumatic fibroma is a reactive oral cavity lesion that clinically manifests as a localized, asymptomatic exophytic papule or nodule of fibrous connective tissue. Traumatic fibroma can result from constant trauma from teeth, restoration, or prosthesis. The most common areas affected by traumatic fibroma include the tongue, mandibular labial, and buccal mucosa. They do not have a risk of malignant transformation. This case report aims to shed light on the unusual clinical appearance of a traumatic fibroma on the dorsum near the tip of the tongue.

## Introduction

Irritation fibroma, also called fibrous hyperplasia, focal fibrous overgrowth, or traumatic fibroma, is a reactive response within the oral cavity. It appears as an exophytic, nonneoplastic, localized inflammatory hyperplastic nodule or papule of connective tissue, This occurs as a result of trauma or irritation. It is a rare benign tumor located near the tip of the tongue, developing due to chronic irritation of the tongue tissues. "Biting fibroma" arises as a result of self-biting [1]. Irritation fibroma is the most common submucosal response to a tooth or prosthetic injury. In 1846, researchers observed the first intraoral fibroma as a polypus and a fibroepithelial polyp [2]. Exophytic, asymptomatic, hard nodules with a pink or smooth surface are typical of irritation fibromas. Nodules often have distinct edges [3]. Other names, such as focal intraoral fibrous nodule, oral polyp, fibrous hyperplasia, and traumatic or irritational fibroma, are known [3].

## Case presentation

A 47-year-old male reported to our department for a routine dental checkup. His history revealed the patient had an episode of accidental tongue-biting four years ago. A general examination revealed his vitals were stale. Intraoral examination revealed a growth near the tip of the dorsum of his tongue. On examination, the growth measured 3 mm × 5 mm in diameter, located near the tip of the dorsum of his tongue, and was ovoid in shape. The surface displayed whitish discoloration due to frequent rubbing with the teeth, causing surface friction (Figure 1).

**Figure 1:**
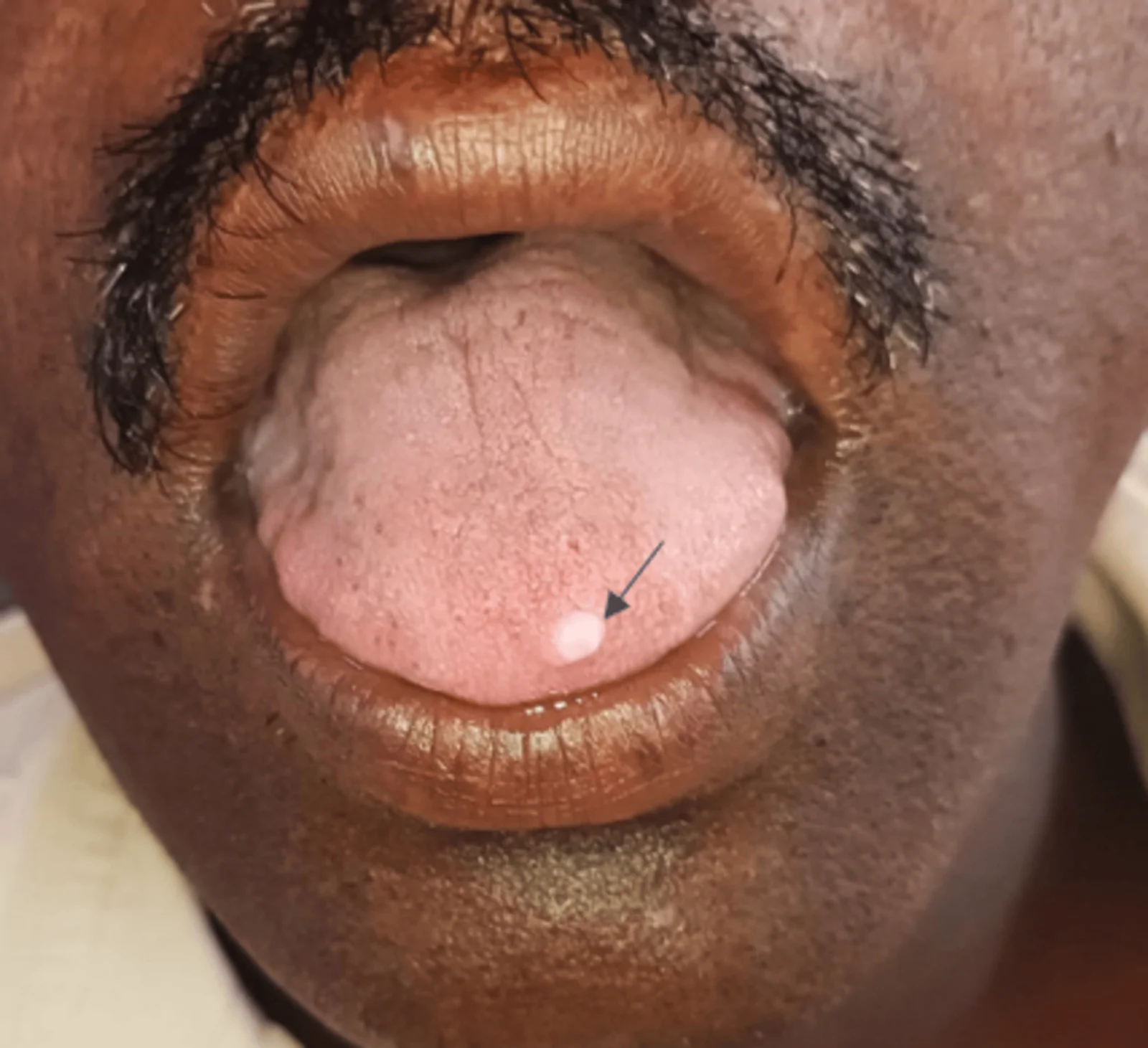
A growth measuring about 3 mm x 5 mm, sessile, and nonpedunculated near the tip on the dorsum of the tongue

No other discomfort, such as pain, is associated with the growth on the tongue. There is no deviation during protrusion of the tongue, no loss of function, no loss of taste sensation, or paresthesia in the tongue associated with the growth. The growth measures about 3 mm × 5 mm in diameter and extends 1 cm away from the tip on the dorsum of the tongue. The growth is firm in consistency, nontender, sessile, and nonpedunculated on palpation. The differential diagnosis includes neuromas, congenital fibrous hamartomas, carcinosarcomas, desmoplastic fibroblastoma, and desmoid-type fibromatosis. The clinical appearance of multiple neuromas differs; traumatic fibromas usually occur solitarily. A congenital fibrous hamartoma is a rare entity with an unknown cause. It is caused by the mandibular arch, which forms the anterior lingual mucosa, not fusing properly. The fibrous structures are displaced along embryonic fusion lines at the tongue tip, either dorsally or ventrally. They appear as either single or multiple masses submucosally near the tip of the tongue [4]. Multiple nodules on the tip and anterior one-third of the tongue occur in multiple endocrine neoplasia type IIb syndrome [5]. Carcinosarcoma tumors involving the tongue mimic traumatic fibromas and clinically appear as exophytic, ulcerated, and polypoid masses in the tongue. Carcinosarcoma is a squamous cell carcinoma variant, which is usually poorly differentiated and composed of biphasic elements, namely sarcomatous and carcinomatous, but has its from squamous epithelium [6]. Desmoplastic fibroblastoma occurs as a painless submucosal mass on the posterior border of the tongue [7]. Desmoid-type fibromatosis clinically presents as a painless submucosal mass up to 2 cm in size in the anterior region of the tongue [8]. Mucoepidermoid carcinoma occurs as a well-circumscribed round mass, measuring 15 mm × 19 mm, on the ventral surface of the right-side tongue near the tip [9]. The patient did not consent to a biopsy, so a histopathological examination was not performed in this case. The patient was informed about the benign nature of the lesion, and the patient was relieved of unwanted apprehension or fear of cancer. No treatment was advocated in our case, as the lesion was asymptomatic. The patient was followed up after a month, and the patient reported no increase in the size of the growth.

## Discussion

"Fibrome en pastille" is a benign (noncancerous) tumor composed of fibrous or connective tissue. The term "en pastille" specifically refers to its characteristic shape and appearance, resembling a small, coin-shaped lesion [10]. The French term "en pastille" means "in tablet" [10]. Fibrome en pastille is usually a tiny, spherical, hard, well-defined skin nodule. These fibromas can occur anywhere; however, they are most common on the arms and legs. Typically, Fibrome en pastille is asymptomatic. In areas with friction or pressure, it may cause discomfort. Medical professionals diagnose it through physical examinations. A biopsy under a microscope can confirm the diagnosis. Fibrome en pastille is benign, so treatment is usually not needed unless it causes symptoms or cosmetic issues. Surgical removal or other minor dermatological procedures may be needed. Fibrome en pastille has no potential for cancer; hence, its prognosis is good [10].

Traumatic or irritational fibroma is an exophytic, common benign reactive lesion of the oral mucosa caused by trauma. Chronic granulation tissue and scarring cause fibroma, a fibrous submucosal tumor. Rarely, repetitive trauma at the same place causes recurrences. There is no risk of cancer in the lesion [11]. Traumatic fibromas are most common in the buccal mucosa, tongue, and mandibular labial mucosa. The lesions have a broad base, are lighter in color than adjacent tissue, and have a white surface due to secondary trauma or hyperkeratosis caused by surface friction [11]. Barker and Lucas found that collagen arrangement in trauma fibromas depends on location and aggravation. Radiating and circular patterns exist. When stress is high, a radiating pattern appears in immovable sections like the palate, whereas a circular pattern appears in flexible areas like the cheeks, with the least trauma [11]. The histological criteria for fibromas by Barker and Lucas include the following: (a) collagen character within the lesion, (b) capsule, and (c) clear demarcation from surrounding tissues [11]. The surface of the fibroma usually appears identical in color to the adjacent mucosa. In our case, the surface appeared whiter in color due to the frequent surface friction or rubbing on the neighboring teeth during activities like eating, chewing, and speaking. Surgical excision of traumatic fibroma using a scalpel and diode laser is straightforward, efficient, and cheap, providing cosmetic and functional clearance. Compared to scalpel knife surgery, diode laser removal causes no bleeding and reduces pain [11]. Table 1 presents the various research studies on case reports on fibroma of the tongue.

**Table 1:** Research studies on case reports of fibroma on the tongue

Author	Year	Age/gender	Clinical findings
Dongre and Khopkar [12]	2011	8-year-old female child	An asymptomatic smooth nodule, single, sessile, firm, pink, and of size 0.5 cm x 0.5 cm on the tongue dorsum for the past 1 year
Asundaria and Tavargeri [13]	2023	11-year-old boy	Enlargement as a spherical mass 9 mm x 10 mm in size on the left side of the tongue for the past three months
Sufiawati et al. [14]	2024	9-year-old female	A single, sessile, exophytic, pink mass, smooth-surfaced lesion measuring 0.8 cm × 0.6 cm × 0.6 cm on the lateral side on the right side of the tongue

## Conclusions

The case enlightens the unusual clinical appearance of the fibroma occurring near the tip of the tongue in the size of the tablet or pastille form. Its white keratotic surface is caused by an accidental trauma caused by biting on the tongue. Irritation fibroma can develop as a result of chronic irritation caused by accidental biting of the cheek or lip. Oral physicians clinically diagnose such fibromas during a routine clinical examination. They do not require surgical intervention unless they create discomfort due to pain or cosmetic disturbances. They do not turn malignant. Counseling is necessary to provide unwanted apprehension or fear of cancer.
